# Effect of different doses of camelina cake inclusion as a substitute of dietary soyabean meal on growth performance and gut health of weaned pigs

**DOI:** 10.1017/S0007114524000722

**Published:** 2024-06-28

**Authors:** Diana Luise, Federico Correa, Giulia Cestonaro, Eleonora Sattin, Giuseppe Conte, Marcello Mele, Ivonne Archetti, Sara Virdis, Clara Negrini, Incoronata Galasso, Claudio Stefanelli, Maurizio Mazzoni, Luigi Nataloni, Paolo Trevisi, Enrico Costanzo

**Affiliations:** 1 Department of Agricultural and Food Sciences (DISTAL), University of Bologna, Viale G Fanin, Bologna 40127, Italy; 2 Cereal Docks S.p.A – Dipartimento Ricerca & Innovazione (E. Costanzo, G. Cestonaro), Cereal Docks S.p.A (L. Nataloni) via Innovazione 1, Camisano Vicentino, 36043, Italy; 3 BMR Genomics, Via della repubblica, Padova, 35131, Italy; 4 Department of Agriculture, Food and Environment (DAFE), University of Pisa, Via del Borghetto, 80, Pisa, 56124, Italy; 5 Istituto Zooprofilattico Sperimentale della Lombardia e dell’Emilia Romagna Bruno Ubertini, V. Bianchi 9, 25124, Brescia, Italy; 6 Institute of Agricultural Biology and Biotechnology, CNR, via Alfonso Corti 12, Milan, Italy; 7 Department for Life Quality Studies, Alma Mater Studiorum, University of Bologna, Corso D’Augusto 237, 47921 Rimini, Italy; 8 Department of Veterinary Science, University of Bologna, Via Tolara di Sopra, 50, 40064, Ozzano dell’Emilia, Italy

**Keywords:** Alternative protein, Microbiota, Feed digestibility, Livestock sustainability, Farm-to-fork

## Abstract

Camelina cake (CAM) is a co-product proposed as an alternative protein source; however, piglet data are still limited. This study aimed to evaluate the effect of different doses of CAM in substitution of soyabean meal on the growth, health and gut health of weaned pigs. At 14 d post-weaning (d0), sixty-four piglets were assigned either to a standard diet or to a diet with 4 %, 8 % or 12 % of CAM. Piglets were weighed weekly. At d7 and d28, faeces were collected for microbiota and polyamine and blood for reactive oxygen metabolites (ROM) and thyroxine analysis. At d28, pigs were slaughtered, organs were weighed, pH was recorded on gut, colon was analysed for volatile fatty acids (VFA) and jejunum was used for morphological and gene expression analysis. Data analysis was carried out using a mixed model including diet, pen and litter as factors; linear and quadratic contrasts were tested. CAM linearly reduced the average daily gain from d0–d7, d0–d14, d0–d21 and d0–d28 (*P* ≤ 0·01). From d0–d7 increasing CAM linearly decreased feed intake (*P* = 0·04) and increased linearly the feed to gain (*P* = 0·004). CAM increased linearly the liver weight (*P* < 0·0001) and affected the cadaverine (*P* < 0·001). The diet did not affect the ROM, thyroxine, intestinal pH, VFA and morphology. All doses of CAM increased the α diversity indices at d28 (*P* < 0·05). CAM at 4 % promoted the abundance of Butyricicoccaceae_UCG-008. Feeding with CAM enhanced resilience in the gut microbiome and can be evaluated as a potential alternative protein source with dose-dependent limitations on piglet growth performance.

The livestock production system has a significant impact on the environment, affecting air, water and soil quality. Feed production is the main contributor, causing climate change and eutrophication^(1)^. It also affects biodiverse regions, especially when feed is sourced from unsustainable areas, contributing to global problems such as deforestation and climate change^(1–3)^. The European Union faces a structural protein deficit and calls for feed diversification and reduced imports of agricultural products, especially soya. Soyabean meal is the main source of protein in pig diets, which is a sustainability issue^(4)^. The development of sustainable protein sources equally able to maintain the pig performance and efficiency is crucial.

Camelina (*Camelina sativa* (L.) Crantz) is a fast-growing annual plant belonging to the *Brassicaceae* family, characterised by a low requirement of inputs including water, fertilizers and pesticides, a great potential for organic production and for sequestering soil nitrate^(5–8)^. Camelina seed can be used to produce edible oil or biofuel, and camelina cake (CAM), obtained as a by-product by cold extraction of the oil fraction, could be used for livestock nutrition due to its high crude protein level (up to 30–35 %) and residual oil content (10–20 %)^(9)^. The residual oil characteristics display promising advantages for egg^(10)^ and poultry meat quality^(11)^. In fact, camelina oil contains about 35 % *α*-linolenic acid^(12)^, a large levels of phytosterols, such as sitosterol (more than 600 mg/100 g) and tocopherol, rendering it a functional feed of interest^(13)^.

The major limitation to the inclusion of CAM in monogastric diets, particularly for younger animal categories, is its anti-nutritional factors. These factors consist of glucosinolate (34·4–36·3 µmol/g) and antitrypsin factor (12–28 TIU/mg) content^(14)^. Contradictory effects are reported on the impact of CAM on the growth performance of broiler. Its dietary inclusion at 8 and 16 % increased body weight (BW) and average daily gain (ADG) of broiler^(15)^. Other studies showed that CAM inclusion from 5 % to 16 % did not affect the growth performance,^(11,15,16)^ and according to the study of Oryschak et al.^(15)^, the dietary inclusion of up to 24 % did not have any adverse effects on the mortality or indicators of toxicity but reduced the feed intake (FI) and the ADG of tin broiler chickens.

For pigs, particularly during the weaning phase, research is extremely limited, and there is insufficient data to make any solid conclusions, particularly with regard to the maximum usage of this raw material. For instance, a decrease in growth performance has been observed in piglets fed diets containing 6 % of CAM in the initial 28 d after weaning^(17)^; similarly, a reduction in ADG and FI was observed in growing finishing pigs fed a 10 % of CAM^(18)^. The authors attributed the negative performances to the low digestibility of CAM. However, according to Ameida et al.^(19)^, the AA standardised digestibility (SID) of camelina expeller is almost comparable to rapeseed meal, around 20 % less than that one of soyabean meal. The low digestibility can be attributed to both the presence of antinutritional factors and high fibre content. While the impact can be marginal in older pigs, which are less susceptible; it is suggested to include CAM in the diet of pigs weighing between 11 and 15 kg of weight (approximately two weeks post-weaning), once their gut has matured enough and their enzymatic profile has fully adapted to a plant-based diet. The digestibility of protein sources may affect gut health as defined by Chalvon-Demersay et al.^(20)^, modulating the gut microbiota and its fermentation activity, the functionality of the gut mucosa and the oxidative and local immune response capacity of pigs. In addition, the high presence of glucosinolates in camelina seeds seems to have a negative effect on the thyroid activity. Indeed, the degradation of these compounds by the myrosinase, an enzyme naturally present in the seed, releases anti-thyroid compounds such as thiocyanate, which in turn can reduce the iodine bioavailability and harm on the piglet’s physiology^(21)^. Therefore, the hypothesis of the present study is that due to the properties of CAM and the physiological gut maturity of pigs at 11–15 kg, it is possible to partially replace soyabean meal with CAM without compromising the physiology, health and performance of weaned pigs.

The aim of the present study is to determine the effect of increasing levels of CAM on the growth performance and gut health of weaned pigs.

## Materials and methods

### Animals, experimental design and diets

At weaning (28 ± 2 d old; d-14), 64 castrated male piglets (7751·8 ± 980 g) were transferred to the University of Bologna’s animal husbandry facility. Upon arrival, the piglets were allocated in thirty-two weaning pens with slatted floors, consisting of two piglets per pen. The piglets were housed in climate-controlled rooms and had continuous access to feed and water. In addition, each pen was equipped with enriched materials (a chain and a natural cotton rope). During the first 2 weeks post-weaning (from d -14 to d0), the pigs were fed the same diet formulated to meet the dietary requirements according to the 2012 NRC^(23)^ recommendations. This 2-weeks adaptation phase was defined based on the results obtained in previous studies that highlighted potential adverse effects on the performance of weaned piglets fed diets containing CAM^(17)^. At d0, the piglets were weighted and arranged into four homogeneous groups balanced for litter of origin and weight. Each group consisted of eight experimental units (pens) formed by two piglets each. Each group was assigned to one of the four experimental diets: (1) standard diet usually used for post-weaning pigs (CO); (2) diet in which 4 % of CAM (C4) was included to replace soyabean meal; (3) diet in which 8 % of CAM (C8) was included to replace soyabean meal and (4) diet in which 12 % of CAM was included to replace soyabean meal. The ingredient and calculated composition of the basal diet (g/kg as fed basis) is reported in Table 1. Moreover, before using the diets, chemical composition, fatty acid profile and amino acid profile of the diets were analysed; results are reported in online Supplementary Table 1 and 2. Accordingly with the aminogram, the AA profile of the diets was adjusted to meet the nutritional requirements reported in the NRC 2012^(10)^ and to be iso-aminoacidic.

**Table 1.** Ingredient and calculated composition of the basal diet (g/kg as fed basis)

Items	Diet*			
	CO	C4	C8	C12
Ingredients, %				
Barley	19·7	19	17·7	19
Flaked barley and corn (50:50)	15	15	15	15
Corn	14	14	14	12
Soyabean meal	11	8·5	6·4	3·5
Camelina cake	–	4	8	12
Wheat flour	10	10	10	10
Wheat	8·2	8	8	8
Bran	6	5	5	5
Lactose	3	3	3	3
Fish meal	2	2	2	2
Soyabean oil	2	2	1·5	1·5
Soya protein concentrate (63 % CP; Soycomil†)	2	2	2	2
Beet pulp	1·5	1·5	1·5	1·5
Oat bran	1	1	1	1
Coconut oil	1	1	1	1
Bicalcium phosphate	1	1	1	1
L-LysineHCL	0·58	0·61	0·63	0·65
Sodium chloride	0·4	0·4	0·4	0·4
Enzymes Premix	0·3	0·4	0·4	0·4
Calcium formate	0·3	0·3	0·4	0·3
L-Threonine	0·25	0·26	0·26	0·24
Amino acid premix:Val, Ile, Leu, His	0·25	0·25	0·25	0·25
DL-Methionine	0·21	0·21	0·21	0·18
Flavouring	0·09	0·09	0·09	0·09
L-Triptophan	0·045	0·05	0·05	0·05
Calculated chemical composition, %				
Dry matter	89·83	89·93	90·04	90·16
Humidity	10·17	10·07	9·96	9·84
Crude protein	16·45	16·47	16·63	16·57
Fats	5·48	5·29	5·58	5·51
Crude fibre	3·44	3·72	4·03	4·42
Ashes	4·38	4·43	4·52	4·60
NDF	14·39	14·92	15·41	16·29
ADF	4·88	5·40	5·95	6·56
ADL	0·82	1·03	1·25	1·49
Starch	40·56	40·29	39·21	38·57
DE Kcal/kg	3378·1	3358·4	3358·0	3325·2
ME Kcal/kg	3241·4	3222·9	3222·8	3191·2
Lys SID	1·13	1·08	1·04	0·98
Met SID	0·44	0·46	0·47	0·44
Trp SID	0·23	0·22	0·20	0·19
Thr SID	0·74	0·70	0·66	0·60
Met + Cist SID	0·70	0·73	0·75	0·74
Val SID	0·76	0·71	0·66	0·61
Ile SID	0·59	0·54	0·50	0·44
Leu SID	1·09	1·01	0·93	0·83
Hist SID	0·37	0·34	0·32	0·29
Arg SID	0·83	0·75	0·67	0·58
Ca	0·69	0·70	0·71	0·72
P	0·71	0·69	0·67	0·66
Analysed glucosinolates mmol/kg dry matter	NA	not detectable	1·53	4·17

* Diet: CO = control diet; C4 = diet with the inclusion of 4 % of CAM; C8 = diet with the inclusion of 8 % of CAM; C12 = diet with the inclusion of 12 % of CAM.

† Soycomil = Commercial product, ADM Animal nutrition, Quincy, USA.

The concentration of the main antinutritional factors including glutaconates and beta mannans^(24)^ was analysed in CAM. Since the beta-mannan concentration in CAM was 0·53 %, a beta-mannanase was included in the diet (Hemicell® HT, Elanco Italia S.p.A, Sesto Fiorentino). Diets were analysed for their content in glucosinolates. For glucosinolate content evaluation, 5 g of each experimental diet (C0, C4, C8 and C12) was defatted two times with hexane (1:10 w/v). The defatted diets were dried and stored at −20°C in fridge until the analysis which were carried out following the procedure described by Pozzo et al.^(25)^.

### Data and samples collections

Piglets were individually weighed on d-14, d0 and weekly until d28 (42 d post-weaning). FI and residue were measured daily to calculate the feed conversion ratio (FCR). Health status was monitored throughout the study; mortality, frequency and type of therapeutic interventions and faecal score (5-score scale: (1) hard and dry; (2) well-formed solid faeces; (3) formed faeces; (4) pasty faeces and (5) watery faeces^(26)^) were recorded daily.

On d7 and d28, a rectal swab was taken from each pig to determine the gut microbial profile using a next-generation technique and the analysis of polyamine concentration. In addition, at the same time points, a blood sample was collected individually using a clot activation tube (Vacutest Kima Srl, Arzergrande PD Italy) and centrifuged at 3000 × g for 10 min after incubation at 37°C for 2 h of to collect serum.

At the end of the trial (d28), all pigs were anaesthetised with Zoletil 100 (Virbac) and then killed by intracardiac injection of 5 ml/kg BW of Tanax ® (embutramide 200 mg/m, mebenzonium iodide 50 mg/ml tetracaine hydrochloride 5 mg/ml; Intervet Productions srl, Aprilia, Italy). The intestinal tract was than isolated, and the contents of the jejunum, caecum and colon were collected and immediately diluted with distilled water in a 2:1 ratio to determine the pH using a pH meter (Vio 7, Sinergica Soluzioni). A second aliquot of the colon was immediately frozen in liquid nitrogen for subsequent analysis of volatile fatty acids (VFA) and lactic acid. In addition, two samples of intestinal mucosa from the distal part of the jejunum were collected; one sample was immediately frozen in liquid nitrogen and stored at −80°C for gene expression analysis, while the other was fixed in paraffin for morphological analysis.

Finally, the weights of kidneys, liver, spleen, empty small intestine and empty colon were recorded. The weight of the organs was expressed as a percentage of the individual pigs’ weight^(27)^.

#### Analysis of microbial profile and intestinal fermentation

Faecal samples were used to extract the total bacterial DNA following the manufacturer’s instructions of the SPIN Kit for Soil (MP Biomedicals). DNA concentration and purity were controlled using a NanoDrop spectrophotometry (Fisher Scientific). DNA samples were then diluted and amplified for the V3-V4 region of the 16S rRNA gene using the Pro341F and Pro805R primers modified with Nextera XT universal tail and the Platinum™ Taq DNA Polymerase High Fidelity (Termo Fisher Scientific). Libraries and sequencing were performed using the MiSeq® Reagent Kit V3, 300PE strategy on the Illumina® MiSeq platform. Microbial data analysis was carried out using the DADA2 pipeline^(28)^, and taxonomy was assigned using the Silva Database (release 138·1) as a reference^(29)^.

The faecal concentrations biogenic amines (putrescine, spermidine and spermine; nmol/ml) were measured using high-performance liquid chromatography and quantified using fluorimetry as described by Pinna et al.^(30)^.

The concentration of VFA and lactose (mg/g) in the colon was obtained using the protocol described by Sandri et al. (2017)^(31)^. Briefly, the caecal content was diluted 1:5 in a 0·1 N solution of H_2_SO_4_, homogenized for 2 min using UltraTurrax (IKA®-Werke GmbH & Co. KG) and centrifuged at 5000 × g for 15 min at 4°C. The supernatant was subsequently filtered using SLMV033RS, 0·45 μm filters (Millex-HV, Merck-Millipore) and injected (20 ul) directly into the high-performance liquid chromatography apparatus using an Aminex HPX-87 H ion exclusion column (300 mm × 7·8 mm; 9 μm particle size; Bio-Rad) kept at 40°C. The detection wavelength was 220 nm. The analyses were performed by applying an isocratic elution (flow rate 0·6 ml/min) with a solution of H_2_SO_4_ 0·008 N as the mobile phase. Individual VFA and lactic acid were identified using a standard solution of 4·50 mg/ml lactic acid, 5·40 mg/ml acetic acid, 5·76 mg/ml propionic acid, 7·02 mg/ml of butyric acid and isobutyric acid, 8·28 mg/ml of valeric acid and isovaleric acid in 0·1 N H_2_SO_4_ (69775, 338826, 402907, B103500, 58360, 75054, 129542, respectively; Sigma-Aldrich). Quantification was performed using an external calibration curve based on the above standards.

### Analysis of the reactive oxygen metabolites and hormones in blood

Serum was analysed colourimetrically for reactive oxygen metabolites (ROM) using the d-ROM test kit (Diacron International Sr1). For the analysis, the serum samples were diluted 1:20 in distilled water and incubated for 5 min at 37°C with a mixture containing 0·01 M acetic acid/buffer sodium acetate pH 4·8 and N,N-diethyl -p-phenylenediamine as a chromogen. Absorbance was read at 520 nm. Each sample was analysed in duplicate^(32)^. Furthermore, serum collected at d28 was used to quantify the concentration of thyroxine (T4) hormone as markers of iodine status and tyroid activity^(33)^ which could had been compromised by the higher glucosinolate concetration in CAM diets. T4 concentration (μg/dl) was analysed colourimetrically on undiluted serum samples using the Immulite/Immulite 1000 Total T4 immunoenzymatic kit using the Immulite® system (Siemens Healthcare Diagnostics Products Limited)^(34)^.

### Analysis of the jejunal gene expression

Total RNA was extracted from each jejunal mucosa using the GENE JET Kit (Thermo Fisher Scientific, Milan) according to the standard protocol. To eliminate any traces of DNA, the samples were treated with DNAase (TURBO-DNASI, Thermo Fisher Scientific). The quality and quantity of RNA were evaluated by spectrophotometric reading of the samples using the Nanodrop instrument. A total of 600 ng of RNA was then converted into complementary DNA using a high-capacity RNA-to-cDNA™ kit (Thermo Fisher Scientific) according to the standard protocol. The complementary DNA was then used for expression analyses by real-time duplex PCR reactions on the Applied Biosystems QuantStudio™ 7 Flex Real-Time PCR system (Thermo Fisher Scientific) using the following programme: 50°C for 2 min, 95°C for 2 min and forty cycles of 95°C for 1 sec and 60°C for 20 sec. Each reaction contained 2 µl of cDNA and 8 µl of a mixture containing primers, probes (online Supplementary Table 3) and 2X TaqMan Mastermix. Each sample was analysed in triplicate. The hydroxymethylbilane synthase (*HMBS*) gene was used as an endogenous gene. The QuantStudio Design and Analysis v2·5 software (Thermo Fisher Scientific) was used to determine gene expression cycle threshold (Ct) values. For each sample, the Ct value of the *HMBS* gene was subtracted from the Ct value of the target gene (ΔCt). The mean ΔCt value of the reference animals was then subtracted from the ΔCt value of all samples (ΔΔCt). Gene expression was calculated using the 2^ΔΔ Ct^ method.

### Analysis of the jejunal morphology

Jejunal tissue samples were fixed in 10 % buffered formalin (pH 7·2) for 48 h and successively embedded in paraffin. Ten sections (7 µm thick) were cut from each paraffin block and mounted on poly-L-lysine slides. Sections were deparaffinised in xylene, rehydrated in decreasing alcohol baths and stained with haematoxylin and eosin. For each sample, the height and width of 20 villi and the width and depth of 20 crypts were measured; only villi and crypts perpendicular to the *muscularis mucosae* were considered valid for morphometry. Sections were evaluated using a 20× objective on an optical microscope (Nikon Eclipse Ni microscope) equipped with a Nikon DS-Qi1Nc digital camera and NIS Elements software BR 4·20·01 (Nikon Instruments Europe BV). Minor adjustments to contrast and brightness were made using Corel Photo Paint, Corel Draw (Corel Photo Paint and Corel Draw).

Villus height was measured as the distance between the villus tip and the point where adjacent intestinal glands (crypts) terminate at the surface of the intestinal mucosa, while crypt depth was measured from the base of the crypt to the level of its opening. The method of Trevisi et al.^(27)^ was used for the evaluation of the absorptive surface of the intestinal mucosa. In this calculation of the absorptive surface, (M) represents the mucosal-serosal amplification ratio. This ratio is calculated using a mathematical formula that takes into account several measurements: the average surface area of the villi (calculated using the length and width of the villus), the mucosal unit (determined by the width of the villus and crypt) and the width of the villus. Based on the previous evaluations, the formula obtained is as follows: M = (villus surface + unit base - villus base)/unit base, where villus surface = π (villus length × villus width), unit base = π (villus width/2 + crypt width/2)^2^ and villus base = π (villus width/2)^2^.

### Statistical analysis

Data on BW, ADG, ROM, T4, organs’ weight, villus height, villus width and crypt width were analysed using a linear mixed model and ANOVA procedure considering the diet (CO, C4, C8, C12) as a fixed factor and the pen and litter as random factors. The polyamines and VFA concentrations and crypt depth, M index, VH:CD ratio data were not normally distributed. For these parameters, a general linear mixed model followed by an ANOVA procedure considering the diet as a fixed factor and the pen and litter as random factors using either a gamma or Poisson distribution and a log transformation was carried out. Data on daily FI and FCR were analysed using a linear model and ANOVA procedure considering diet as the factor and the pen as the experimental unit. Subsequently, the linear and quadratic polynomial contrasts for the diet were carried out. *P* values ≤ 0·05 were considered statistically significant, while *P* values ≤ 0·1 were considered a trend of significance.

For the microbial profile, *α* (Shannon, Chao1 and InvSimpson indices) and β diversity (calculated as weighted Unifrac distance matrix), as well as the abundance of taxonomic categories, were analysed with the R software 3·6 using the PhyloSeq^(35)^, Vegan^(36)^ and microbiomeMarker^(37)^ packages. The *α* diversity indices were analysed using an ANOVA model (lm function) including diet a fixed factor and the pen and litter as random factors. Furthermore, linear and quadratic contrasts were carried out to assess the effect of CAM supplementation. The effects on β diversity were visualised using a non-metric multidimensional scaling approach (plot_ordination function). The analysis was carried out in the dataset composed of each single time point (d7 and d28). Linear discriminant analysis (LDA) effect size algorithm at genus level was applied to identify taxa differentially expressed (LDA score > 3 and *P*.adj < 0·05) between the dietary groups. In addition, the data obtained from 16S microbial characterisation were used for the functional prediction of microbiome metabolism. This prediction was performed using the Tax4Fun2 package^(38)^. Functional profiles were predicted by aligning the 16S sequence of each taxa with the reference dataset to identify the nearest neighbours. The metabolic potential was then calculated by associating the sequence abundance with KO databases (Kyoto Encyclopedia of Genes and Genomes). The abundance of metabolic pathways was then normalised to the number of copies of the 16S rRNA gene of each taxon. Finally, the effect of the diet on the microbial functional profile was analysed using the LEfSe algorithm (LDA score > 2 and *P*.adj < 0·05).

## Results

### Effect of camelina cake on growth performance and health

During the *in vivo* study, the animals were in good health, and no pharmacological treatments were required. Table 2 shows the effect of CAM inclusion on the growth performance of the pigs. At d7 and d14, no difference in BW was observed; at d21, the diet did not affect the BW of the pigs, but a trend for a linear decrease in BW with the increase in CAM was observed (*P* = 0·06). At d28, the end of the study, a trend (*P* = 0·10) of the diet and a linear decrease in BW with the increase in CAM were observed (*P* = 0·05).

**Table 2.** Effect of camelina cake on the growth performance of post-weaning piglets

Item	Diet*				s em	*P*	Contrasts *P*	
	CO	C4	C8	C12		Diet	Linear	Quadratic
Body weight, g								
d0	11 562	11 563	11 563	11 563	530	1·00	1·00	1·00
d7	14 918	14 119	14 186	13 922	595	0·44	0·16	0·56
d14	19 133	18 376	18 425	17 728	720	0·41	0·13	0·96
d21	24 620	23 431	23 693	22 325	907	0·19	0·06	0·91
d28	29 669	27 922	28 871	26 539	1043	0·10	0·05	0·76
Average daily gain, g/d								
d0–d7	480	366	376	338	24·2	< 0·0001	0·0004	0·11
d7–d14	604	610	607	545	35·4	0·49	0·26	0·33
d0–d14	542	488	491	441	26·7	0·04	0·01	0·93
d14–d21	784	722	752	656	40	0·12	0·05	0·66
d0–d21	623	566	578	513	27	0·03	0·01	0·87
d21–d28	719	640	738	623	44·4	0·17	0·34	0·69
d14–d28	751	681	745	628	37·5	0·06	0·08	0·54
d7–d28	703	657	699	601	30·9	0·07	0·07	0·39
d0–d28	647	585	619	535	25·9	0·02	0·01	0·69
Feed intake g/d								
d0–d7	674	598	624	579	27·50	0·10	0·04	0·58
d7–d14	941	941	929	856	49·80	0·56	0·23	0·47
d0–d14	808	769	777	718	34·70	0·34	0·10	0·77
d14–d21	1183	1143	1159	1044	53·10	0·29	0·10	0·49
d0–d21	933	894	904	826	37·40	0·25	0·08	0·61
d21–d28	1300	1164	1249	1112	72·90	0·28	0·15	1·00
d14–d28	1241	1154	1204	1078	59·60	0·26	0·11	0·75
d7–d28	1141	1083	1112	1004	51·40	0·29	0·11	0·64
d0–d28	1025	961	990	898	42·30	0·21	0·07	0·73
Feed conversion ratio								
d0–d7	1·42	1·65	1·66	1·74	0·07	0·02	0·004	0·26
d7–d14	1·56	1·55	1·53	1·57	0·04	0·93	0·89	0·61
d0–d14	1·49	1·58	1·58	1·63	0·04	0·12	0·02	0·72
d14–d21	1·52	1·59	1·54	1·60	0·04	0·42	0·29	0·92
d0–d21	1·50	1·58	1·56	1·61	0·03	0·13	0·04	0·66
d21–d28	1·82	1·83	1·71	1·84	0·07	0·54	0·83	0·44
d14–d28	1·66	1·70	1·62	1·72	0·05	0·42	0·64	0·47
d7–d28	1·63	1·65	1·59	1·66	0·04	0·55	0·81	0·50
d0–d28	1·59	1·64	1·60	1·67	0·03	0·31	0·20	0·80

* Diet: CO = control diet; C4 = diet with the inclusion of 4 % of CAM; C8 = diet with the inclusion of 8 % of CAM; C12 = diet with the inclusion of 12 % of CAM.

Regarding ADG, for the periods d0–d7, d0–d14, d0–d21 and d0–d28, the increase in CAM inclusion in the diet leads to a linear reduction in the ADG (*P* < 0·05). For the period d14–d21, the diet did not show significant effects, but the linear contrast showed a reduction of the ADG with the increase in CAM (*P* = 0·05). No differences were observed between the groups for the ADG in the periods d7–d14 and d21–d28.

For the period d0–d7, the diet tended to influence the FI of pigs (*P* = 0·10) and the FI was linearly reduced with the increase in CAM in the diet (*P* = 0·04). No differences due to the diet were recorded for FI in the following weeks, but a trend for a linear reduction in FI with the increase in CAM was observed in the periods d0–d14, d14–d21, d0–d21 and d0–d28 (*P* < 0·10).

The diet affected the FCR for the period d0–d7, (*P* = 0·02), and a linear increase in FCR with the increase in the CAM inclusion in the diet was observed (*P* = 0·004). No differences due to diet were recorded for FCR in the subsequent periods, but a linear increase in FCR with the increase in CAM was observed in the periods d0–d14 and d0–d21 (*P* < 0·05).

### Effect of camelina cake on microbial profile and intestinal fermentations

**Fig. 1.**
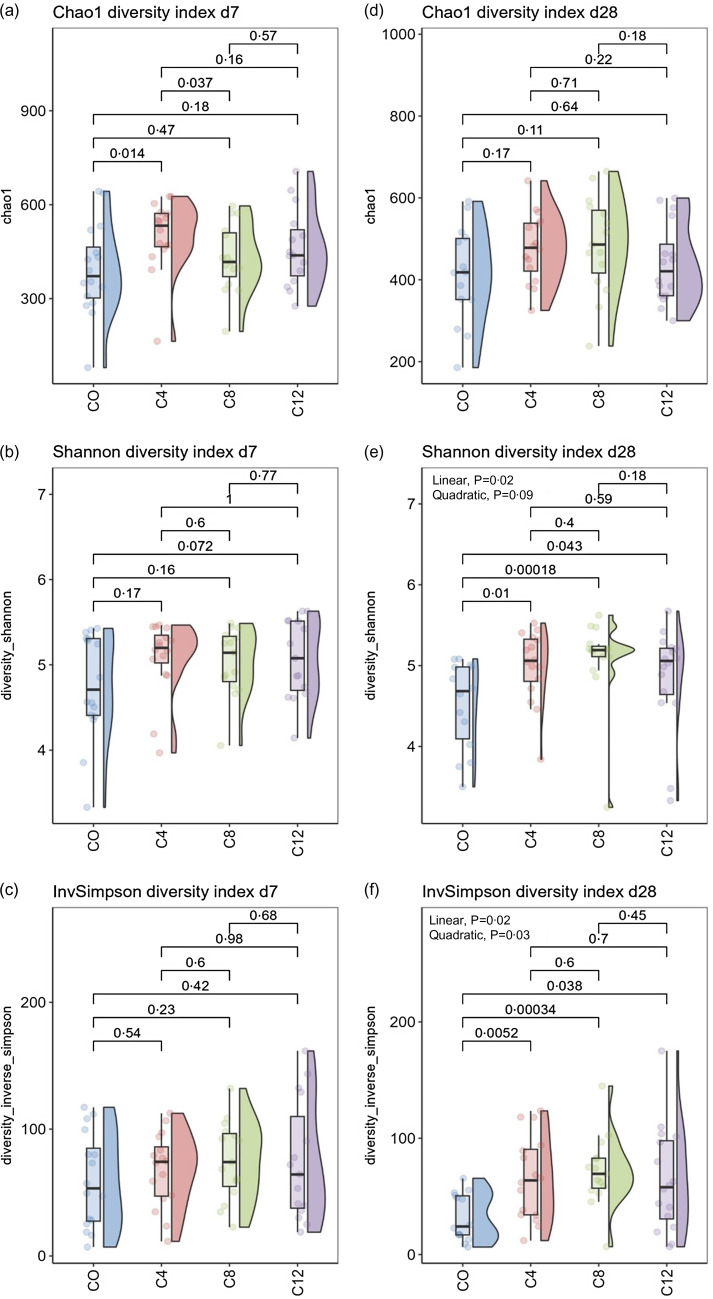
Effect of camelina cake on the *α* diveristy indices in faecal samples of of post-weaning piglets. Diet*: CO = control diet; C4 = diet with the inclusion of 4 % of CAM; C8 = diet with the inclusion of 8 % of CAM; C12 = diet with the inclusion of 12 % of CAM.

Bacterial DNA was successfully extracted and amplified from a total of 126 samples. Overall, the sequencing procedure produced an average of 73 507 sequences per sample; after quality control, an average of 39 959 sequences were retained, which, after bioinformatic analysis, produced a total of 6541 amplicon sequence variants. The rarefaction curves showed that the number of different species, observed as a function of the number of sequences, ranched a plateau trend indicating that the sequencing procedure was able to capture all the variability present in the samples (online Supplemetary Fig. 1).

Among the 6541 recovered amplicon sequence variants, 23 phyla, 102 families, and 241 genera were identified. The most abundant phyla were Firmicutes 64·89 ± 10·9 %, Bacteroidota 24·95 ± 10·3 % and Spirochaetota 4·47 ± 4·88 %. The most abundant families were Lachnospiraceae 15·81 ± 9·11 %, Prevotellaceae 12·72 ± 7·22 %, Oscillospiraceae 8·52 ± 4·18 % and Ruminococcaceae 6·71 ± 3·03 %. The most represented genera were *Lactobacillus* (6·25 ± 11·50 %), *Blautia* (3·96 ± 5·06 %), *Clostridium*_sensu_stricto_1 (5·23 ± 6·95 %) and *Prevotella* (6·33 ± 6·61 %).

The results for the *α* diversity indices at d7 (A, B and C) and d28 (D, E and F) are shown in Fig. 1. At d7, the C4 diet had a higher Chao1 index compared with the CO diet (*P* = 0·014), and the C12 diet tended to have a higher Shannon index compared with the CO diet (*P* = 0·07). No differences were observed for the InvSimpson index at d7. No linear or quadratic effects were observed for any of the indices at d7.

At d28, the CO diet had a lower Shannon and InvSimpson indices compared with the C4, C8 and C12 diets (Shannon: *P* = 0·01; *P* < 0·0001; *P* = 0·04; InvSimpson: *P* = 0·005; *P* < 0·0005; *P* = 0·038). Furthermore, a quadratic effect (*P* = 0·020) and a trend for a linear effect (*P* = 0·09) were observed for the Shannon index and a linear (*P* = 0·02) and quadratic (*P* = 0·03) effects were observed for the InvSimpson index. No differences were observed for the Chao index at d28.

**Fig. 2.**
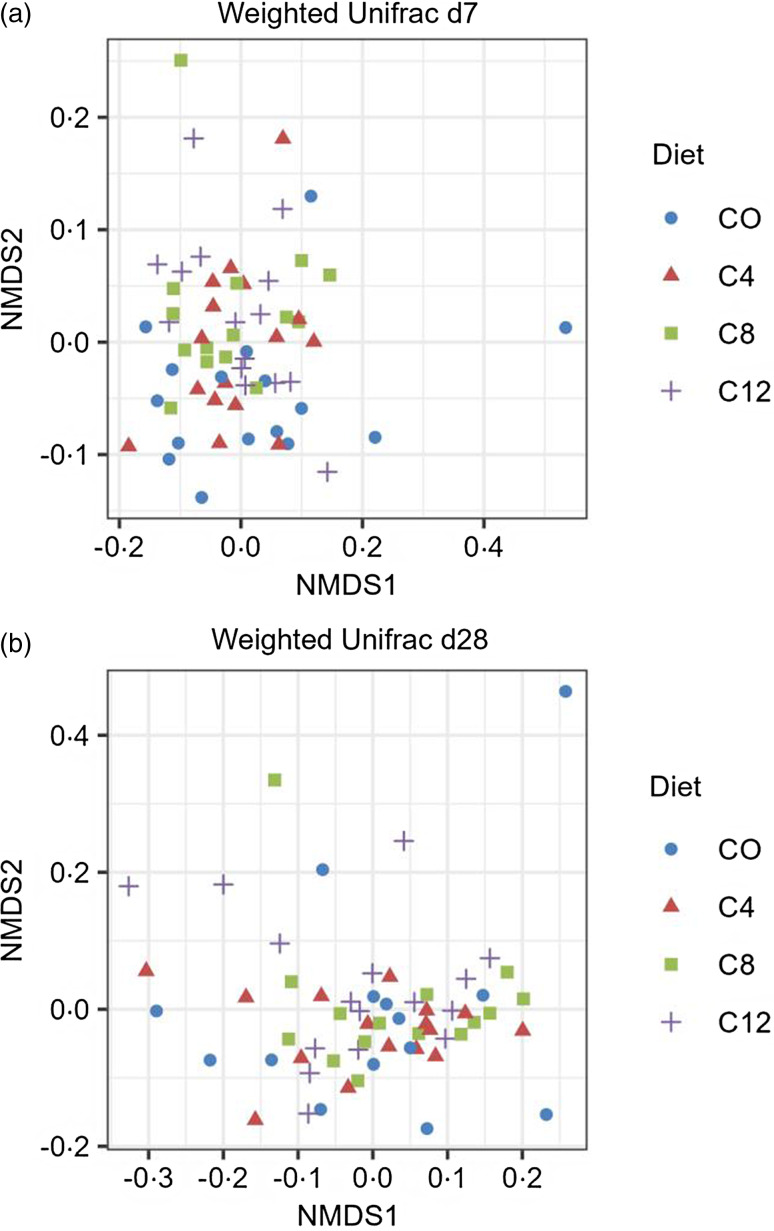
Effect of camelina cake on the β diveristy indices in faecal samples of of post-weaning piglets. Diet*: CO = control diet; C4 = diet with the inclusion of 4 % of CAM; C8 = diet with the inclusion of 8 % of CAM; C12 = diet with the inclusion of 12 % of CAM.

For β diversity, two non-metric multidimensional scaling plots were generated using a weighted unifrac distance matrix for the data at d7 and d28 (Fig. 2). The plots show that the samples from the different diets did not form separate clusters, indicating that the overall microbial composition of the experimental diets was similar. No differences due to the diet on β diversity were observed by the Adonis test (d7: R2 = 0·06, *P* = 0·21 and d28 R2 = 0·05, *P* = 0·36).

**Fig. 3.**
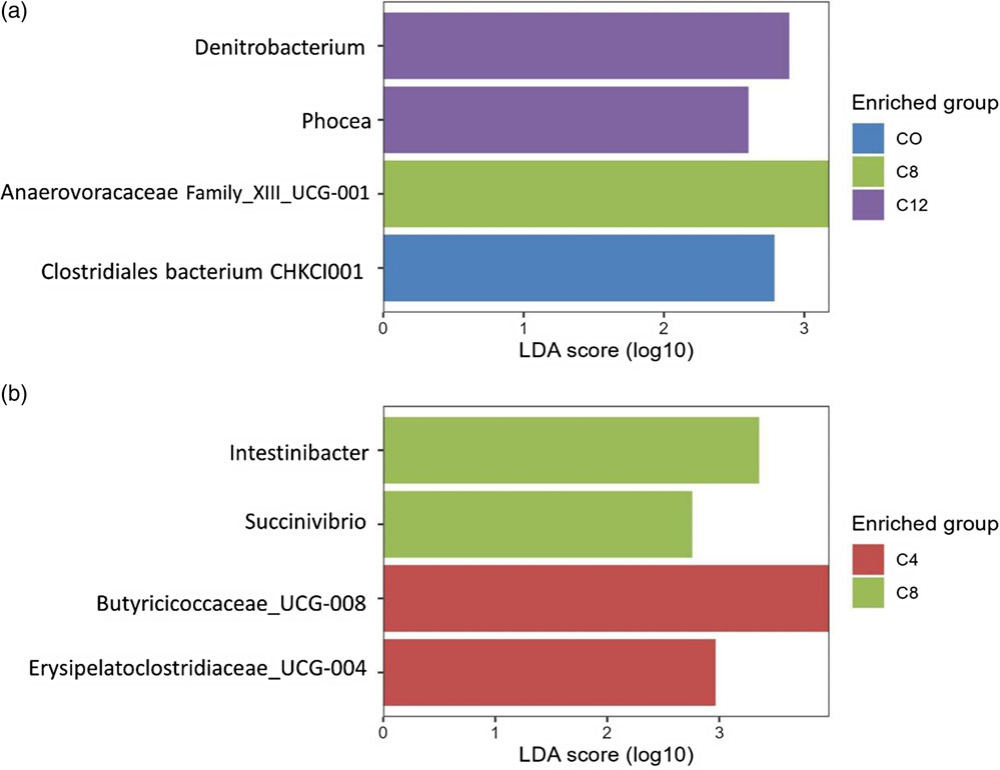
Linear discriminant analysis effect size (LEfSe) plots of the biomarkers taxa, identified in piglets faecal samples at d7 (a) and d28 (b) fed different doses of camelina cake. Diet*: CO = control diet; C4 = diet with the inclusion of 4 % of CAM; C8 = diet with the inclusion of 8 % of CAM; C12 = diet with the inclusion of 12 % of CAM.

To identify specific bacterial markers for each diet, the LEfSe analysis was performed at both d7 and d28 (Fig. 3). At d7, the C0 diet was characterised by a higher abundance of *Clostridiales bacterium* CHKCI001 (LDA score = 2·83, P.adj = 0·03), the C8 diet by a higher abundance of Anaerovoracaceae Family_XIII_UCG-001 (LDA score = 3·20, P.adj = 0·03) and the C12 diet by a higher abundance of *Denitrobacterium* (LDA score = 2·85, P.adj = 0·03) and Phocea (LDA score = 2·58, P.adj = 0·03). At d28, the C4 diet was characterised by a higher abundance of Butyricicoccaceae_UCG-008 (LDA score = 3·98, P.adj = 0·02) and Erysipelatoclostridiaceae_UCG-004 (LDA score = 2·94, P.adj = 0·003), while the C8 diet was characterized by a higher abundance of *Intestinibacter* (LDA score = 3·35, P.adj = 0·03) and *Succinivibrio* (LDA score = 2·74, P.adj = 0·01).

The results of the prediction of functional profiles allowed the identification of 325 metabolic pathways at d7 and 329 at d28. The main pathways were relate to carbohydrate metabolism, membrane transporters, amino acid metabolism, energy metabolism, signal transduction, vitamin metabolism, quorum sensing, nucleotide metabolism, translation and replication and repair. None of the predicted metabolic pathways showed significant differences in terms of abundance among the different diets.

Table 3 shows the effect of the different doses of CAM on the intestinal pH, the faecal concentration of polyamines and the colon concentration of VFA. The pH of none of the intestinal contents analysed was affected by the diet, and no linear or quadratic effects were observed. At d7, the faecal concentration of putrescine was not affected by the diet, but the linear contrast showed a trend of linear increase with the reduction in CAM in the diet (*P* = 0·07); the cadaverine concentration was affected by the diet (*P* = 0·001), showing both a linear and a quadratic effect of CAM concentration (*P* = 0·001), a high concentration in the CO group, followed by C12, C8 and C4 groups was observed. The faecal concentration of spermidine and spermine was not affect by the diet at d7. At d28, none of the poliamines were affected by the diet. The colon concentrations of lactic, acetic, propionic, isobutyric, butyric, isovaleric and valeric acids were not affected by the diet.

**Table 3.** Effect of camelina cake on the intestinal pH, faecal concentration of polyamines and colon concentration of volatile fatty acids of post-weaning piglets

Item	Diet*				sem	*P*	Contrasts *P*	
	CO	C4	C8	C12		Diet	Linear	Quadratic
pH								
Jejunum	7·03	7·04	7·00	7·10	0·08	0·87	0·69	0·59
Caecum	5·92	5·93	5·89	5·82	0·08	0·77	0·36	0·63
Colon	6·41	6·47	6·35	6·37	0·12	0·9	0·65	0·87
Polyamines faeces d7, nmol/g								
Putrescine	108·4	97·2	69·4	75·8	17·25	0·22	0·07	0·55
Cadaverine	150	66·4	86·4	95·3	3·20	<0·001	<0·001	<0·001
Spermidine	378	331	312	387	41·1	0·50	0·96	0·14
Spermina	54·3	27·6	24·5	44·6	29·08	0·68	0·76	0·23
Polyamines faeces d28, nmol/g								
Putrescine	77·5	70·5	71·2	71·9	11·25	0·96	0·49	0·53
Cadaverine	23·6	15·8	30·8	26·7	9·84	0·59	0·51	0·71
Spermidine	236	175	207	208	11·8	0·11	0·49	0·09
Spermina	18·1	15·9	16·1	17·2	2·07	0·84	0·77	0·39
Volatile fatty acids in colon d28 mg/g								
Lactic acid	2·59	2·46	2·91	3·16	0·51	0·70	0·30	0·70
Acetic acid	1·62	2·07	1·41	1·40	0·36	0·52	0·37	0·55
Propionic acid	1·04	1·02	1·09	1·16	0·21	0·89	0·49	0·72
Iso-butyric acid	0·0015	0·017	0·032	0·045	0·02	0·93	0·58	0·76
Butyric acid	3·32	3·79	3·44	3·62	0·78	0·97	0·77	0·77
Iso-valeric acid	1·10	1·32	0·90	1·23	0·31	0·75	0·96	0·81
Valeric acid	0·25	0·19	0·22	0·16	0·15	0·97	0·79	0·97

* Diet: CO = control diet; C4 = diet with the inclusion of 4 % of CAM; C8 = diet with the inclusion of 8 % of CAM; C12 = diet with the inclusion of 12 % of CAM.

### Effect of camelina cake on organs and tissue weight

Table 4 reports the effect of CAM on the weight of organs expressed as a percentage of body weight. The inclusion of CAM in the diet influenced liver weight (*P* < 0·0001), which increased linearly with the increase in CAM inclusion in the diet (linear contrast, *P* < 0·0001). In addition, the diet influenced small intestine weight (*P* = 0·01), and a quadratic effect of CAM inclusion (*P* = 0·03) with lower relative weight in the C4 diet was observed. Regarding the large intestine, colon and caecum were weighted; for the colon weight, a linear trend of increasing weight was observed with the increasing inclusion of CAM in the diet (linear effect, *P* = 0·10). No effects of CAM inclusion were observed for spleen, kidney, and cecum weight.

**Table 4.** Effect of camelina cake on the organs weight as percentage of their body weight of post weaned piglets

Item	Diet*				sem	*P*	Contrasts *P*	
	CO	C4	C8	C12		Diet	Linear	Quadratic
Organs weigh/ body weight, %								
Liver	3·42	3·57	4·03	4·21	0·13	<0·0001	<0·0001	0·74
Spleen	0·28	0·29	0·27	0·31	0·03	0·85	0·65	0·64
Kidneys	0·64	0·64	0·62	0·61	0·02	0·65	0·30	0·76
Small intestine	3·08	2·78	3·07	3·18	0·09	0·01	0·15	0·03
Colon	1·32	1·27	1·40	1·39	0·05	0·12	0·10	0·67
Caecum	0·23	0·23	0·22	0·22	0·01	0·94	0·56	0·84

* Diet: CO = control diet; C4 = diet with the inclusion of 4 % of CAM; C8 = diet with the inclusion of 8 % of CAM; C12 = diet with the inclusion of 12 % of CAM.

### Effect of camelina cake on reactive oxygen metabolites and hormones in blood

Table 5 shows the effect of CAM inclusion on the serum concentration of ROM at d7 and d28 and on the concentration of T4 at d28. No effect of the diet was observed for the ROM concentration at either d7 or d28; however, a trend of a linear effect relative to the reduction in ROM with the increase in CAM inclusion in the diet was observed at d7 (*P* = 0·09). At d28, the concentration of T4 was not affected by the inclusion of CAM in the diet.

**Table 5.** Effect of camelina cake on reactive oxygen metabolites and thyroxine of post-weaning piglets

Item	Diet*				sem	*P*	Contrasts *P*	
	CO	C4	C8	C12		Diet	Linear	Quadratic
ROM, mmol H_2_O_2_/L								
d7	23·90	22·90	20·70	21·20	1·54	0·29	0·09	0·58
d28	27·30	25·80	23·40	24·00	1·78	0·33	0·11	0·50
T4, μg/dl								
d28	5·39	5·74	5·42	5·49	0·34	0·87	0·68	0·48

* Diet: CO = control diet; C4 = diet with the inclusion of 4 % of CAM; C8 = diet with the inclusion of 8 % of CAM; C12 = diet with the inclusion of 12 % of CAM.

### Effect of camelina cake intestinal gene expression and morphology

**Fig. 4.**
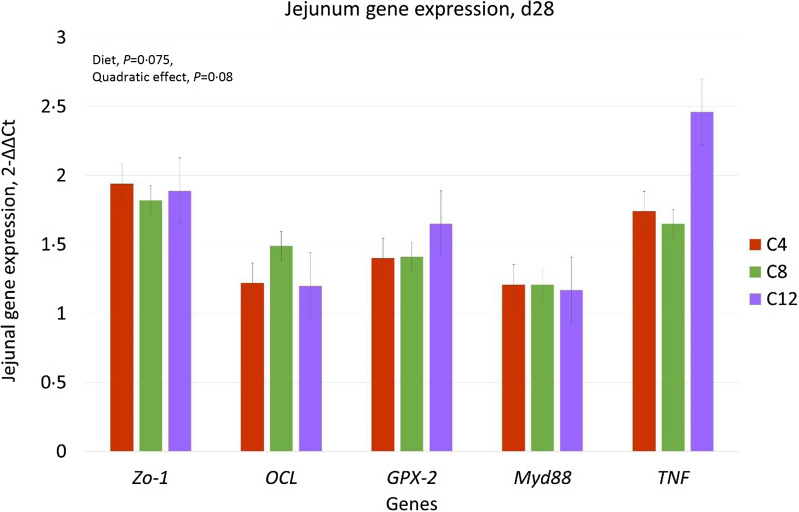
Effect of camelina cake on the jejunual gene expression of post-weaning piglets. Diet: C4 = diet with the inclusion of 4 % of CAM; C8 = diet with the inclusion of 8 % of CAM; C12 = diet with the inclusion of 12 % of CAM.

Figure 4 shows the results of the expression values of the genes zonulin (*ZO-1*), occludin (*OCL*), glutathione peroxidase 2 (*GPX2*), innate immune signalling adaptor (*Myd88*) and *TNF* in the jejunum of piglets fed with the different diets based on CAM compared with piglets fed with the CO diet. The diet tended to influence the expression of *ZO-1* (*P* = 0·075), and a quadratic trend (quadratic contrast, *P* = 0·08) of the effect of CAM inclusion in the diet on *ZO-1* expression was observed, with lower levels in the C8 group. Gene expression of the other genes was not influenced by the diet.

The effect of CAM inclusion on the morphology parameters analysed at d28 is shown in Table 6. None of the analysed parameters showed an effect of the diet, either a linear or quadratic significant contrast.

**Table 6.** Effect of camelina cake on the jejunal morphology parameters of post-weaning piglets

Item†	Diet*				sem	*P*	Contrasts *P*	
	CO	C4	C8	C12		Diet	Linear	Quadratic
Villus height, µm	276	263	252	280	12·0	0·28	0·95	0·08
Villus width, µm	101·3	96·7	92·2	96·5	4·6	0·27	0·20	0·18
Crypt width, µm	172	154	170	170	9·3	0·14	0·70	0·15
Crypt depth, µm	40·0	37·7	38·9	39·2	13·8	0·99	0·97	0·91
M index	6·08	6·09	5·80	6·34	0·37	0·76	0·79	0·45
VH:CD ratio	1·60	1·66	1·44	1·61	0·15	0·37	0·65	0·50

* Diet: CO = control diet; C4 = diet with the inclusion of 4 % of CAM; C8 = diet with the inclusion of 8 % of CAM; C12 = diet with the inclusion of 12 % of CAM.

† Item: M index = index for the mucosa absorption capacity (villous surface + unit bottom − villous bottom)/unit bottom, where villous surface = π (villous length × villous width), unit bottom = π (villous width/2 + crypt width/2)^2^ and villous bottom = π (villous width/2). VH:CD ratio = villus height/crypt depth.

## Discussion

The results of the present study showed that the CAM inclusion in the diet of pigs from three weeks post-weaning can reduce their ADG. This effect was more visible during the first week of supplementation, while it is less evident in the following weeks. However, considering the total duration of the trial, the reduction in ADG in the initial period may led to a global reduction in the ADG in long term. Our results agree with previous studies in which the authors observed decreases in ADG with the inclusion of 6 % and 10 % of CAM in post-weaning^(17)^ and growing pigs^(18)^, respectively. In both these two studies, the authors observed a decrease not only in the ADG but also in the FI and an increase in FCR. The decrease in the FI and the increase in FCR were associated with the higher presence of antinutritional factors, including glucosinolates and trypsin inhibitors, which can reduce the nutrient digestibility, alter the pig physiology and reduce the palatability due to the bitterness^(14)^. On the contrary, in the present study, the inclusion of CAM only impaired the FI during the first week of supplementation. However, an increase in the FCR was observed over a longer period (d0–d21), possibly due to an increase in dietary fibre with the inclusion of the CAM in the diet as well as to the lower AA digestibility of CAM compared to soyabean meal. Indeed, in this study, the CAM diets were supplemented with synthetic AA to achieve the same level of total AA as the control diet, but without considering the lower digestibility of the AA present in CAM itself. Although the digestibility was not analysed in the present study, previous studies reported a low digestibility of crude protein (0·65 – 0·70 SID CP) and AA including Lys (0·58 – 0·80 SID Lys), Met (0·53 – 0·80 SID Met) and Thr (0·53 – 0·66 SID Thr) in CAM in growing pigs^(19,39)^. Furthermore, CAM is characterised by a high presence of glucosinolates and trypsin inhibitor factors^(19)^, which can affect the FCR in pigs. The results of the analysis of the glucosinolates concentration performed on the pure CAM and on the different diets with the CAM inclusion are in line with the expected concentration reported in the literature^(39,40)^ and with the dose of CAM included in the diets.

Camelina seeds are characterised by the presence of aliphatic glucosinolates: 9-(methylsulfinyl)nonyl glucosinolate (also known as glucoarabin or GS9), 10-(methylsulfinyl)decyl glucosinolate (also known as glucocamelinin or GS10) and 11-(methylsulfinyl)undecyl glucosinolate (also known as homoglucocamelinin or GS11)^(41,42)^. Depending on their nature, glucosinolates can either be hydrolysed by myrosinases which can be produced by gut microorganisms^(43)^ producing progoitrin which is spontaneously converted into goitrin at neutral pH and into nitriles at a more acidic pH (< 5·5)^(44)^, or they can be degraded into isothiocyanates and then to indole-3-carbinol by the release of thiocyanate at neutral pH, and to indole-3-acetonitrile at acidic pH (4–5·6)^(45,46)^. Thiocyanate and goitrin can impair the transport of iodine to the thyroid and interfere with the thyroid hormones synthesis which are involved in energy metabolism^(47)^ and observable by a reduction in the blood concentration of T4^(48)^. To evaluate this hypothesis, the T4 blood concentration was analysed, and no difference due to the CAM inclusion was observed. The stability of T4 could be associated with the low level of glucosinolates that leads to low production of their metabolites. Another explanation could be associated with the produced metabolites, which rely on the types of glucosinolates and the intestinal pH. It could be argued that aliphatic glucosinolates in CAM were mainly transformed into nitriles due to the acidic stomach pH of pigs or into goitrogenic compounds in the distal part of the small intestine and large intestine, where the pH is nearly neutral. This transformation potentially led to increased liver activity. It is widely known that dietary glucosinolates can boost metabolic activity and require increased energy from the liver and kidneys to eliminate these compounds. In our study, we observed a proportional increase in liver weight corresponding to the rise in dietary CAM inclusion.

This result is in agreement with previous studies carried out in pigs^(17,18,49)^ fed with CAM and confirms the effect of CAM on the metabolic activity of the liver. Furthermore, it cannot be excluded that the increase in liver weight is also linked to the trypsin inhibitor activity, as reported previously^(50,51)^.

Regarding intestinal health parameters, the results obtained from the present study showed that the inclusion of CAM does not lead to potential risks in growing pigs. Indeed, no differences were observed among the groups for the parameters of intestinal morphology and the expression of genes related to inflammation and intestinal barrier integrity. Only a trend towards higher expression of *ZO-1*, with a quadratic effect of CAM was observed. The *ZO-1* gene encodes a member of the membrane-associated guanylate kinase protein family, which acts as a tight junction protein, involved in the passage of ions and macromolecules between intestinal endothelial and epithelial cells. Therefore, the observed result for *ZO-1* expression suggests that CAM intake may slightly modulate the expression of genes involved in the intestinal barrier. This effect could be due to the high concentration of PUFA and antioxidant and autoinflammatory factors, including polyphenols and flavonoids, in which CAM is rich^(5,52)^. An additional explanation could be related to the fact that the CAM diets were richer in fibre, which could have provided additional benefits for intestinal health^(5)^. The effect on the gut barrier could also be related to a change in the intestinal microbiota in response to the inclusion of CAM in the diet. Indeed, the faecal microbial profile observed in the present study showed that CAM can increase the *α* diversity indices, especially after 28 d of feeding. No comparable studies in pigs were found in the literature to compare the data obtained; however, a similar effect has been reported in the rumen microbiota of lactating cows fed with CAM^(53)^. An increase in *α* diversity values is considered an indicator of a mature microbial community. In fact, higher diversity is associated with greater functional redundancy, which contributes to better stability of the microbial ecosystem in counteracting stressful events that can lead to dysbiotic conditions^(54)^. In addition, the CAM inclusion promoted the abundance of some bacterial markers; among these, the genera Butyricicoccaceae_UCG-008 and Erysipelatoclostridiaceae_UCG-004 are notable. Both have been identified as microbial markers for the C4 diet. These genera are members of the normal porcine intestinal microbiota^(55,56)^. The genera belonging to the family Butyricicoccaceae are considered to be beneficial to health, due to their production of butyric acid. The role of the genera in the Erysipelatoclostridiaceae family is still unclear in the literature. In humans, an excess in the abundance of some genera of this family seems to be associated with intestinal disorders such as Crohn’s disease and *Clostridium difficile* infection^(57)^. However, in pigs, an increase in genera belonging to the Erysipelatoclostridiaceae family was observed with the administration of probiotics^(58)^ and free amino acids^(56)^. Although the inclusion of CAM modified the indices of α diversity and the abundance of specific genera, no significant differences were observed with regard to the structure of the microbial community (β indices) and the metabolic functions of the microbiota (pathway predictions), suggesting that the diet did not radically alter intestinal microbial activity and metabolism. This result is in agreement with the lack of difference in the intestinal pH and colonic VFA concentrations.

Furthermore, since the substitution of soyabean meal by CAM led to a different inclusion of free AA and a different digestibility and fermentation of AA in the gut, the concentration of polyamines in the faeces was analysed. In fact, the faecal concentration of polyamines derives mainly from the microbial decarboxylation of AA by the microbiota in the large intestine and from the absorption capacity of AA and the polyamines in the small intestine^(59)^. At d7, the inclusion of CAM reduced the concentration of cadaverine and tended to reduce the concentration of putrescine in the colon indicating a lower protein fermentation in the large intestine or a higher polyamine absorption in the small intestine. However, the present results could also be explained by the lower FI of the CAM groups, which were lower in the first week. At d28, when the FI was stabilized among the diets, the diet had a quadratic effect on spermidine; spermine is derived from the activity of the enzyme spermidine synthetase starting from putrescine, which, in turn is derived from ornithine via ornithine decarboxylase. Spermidine can also be converted into spermine^(60)^. The quadratic reduction of spermidine in the diet with CAM, not associated with a simultaneous increase in spermine and a reduction in putrescine, may be due to a lower capacity of the intestinal microbiota to convert putrescine into spermidine or to a greater intestinal absorption capacity of spermidine in pigs fed the C4 diet.

### Conclusion

The inclusion of CAM in pig diets from three to eight weeks post-weaning did not affect the intestinal health and the gut oxidative status; however, its inclusion from 4 to 12 % linearly reduced the animals’ ADG, especially during the first week of administration, an effect that was not fully recovered in the following weeks. The reduction in ADG was not attributable to a reduction in FI, for which the effect was observed only in the first week, but mainly to a reduction in feed conversion capacity. It is not excluded that diets containing CAM supplemented with synthetic AA at doses that also consider the reduced digestibility of AA in CAM could mitigate potential negative performance effects. The presence of antinutritional factors did not compromise the synthesis of thyroid hormone, which is involved in the metabolic processes necessary for growth. However, they may have contributed to an increase in the metabolic activity and in the energy requirement of the liver, whose weight increased linearly with the increase in CAM in the diet. In order to promote a wider inclusion of CAM in pig diets, it is suggested to treat the product through technological processes to improve its nutritional composition and digestibility.

## Supporting information

Luise et al. supplementary material Luise et al. supplementary material

## Supplementary material

For supplementary material accompanying this paper visit https://doi.org/10.1017/S0007114524000722
