# Supplementary material for: Effect of different doses of camelina cake inclusion as a substitute of dietary soyabean meal on growth performance and gut health of weaned pigs
Source: Br J Nutr. 2024 Apr 12;131(12):1962–74. doi: 10.1017/S0007114524000722 (PMC11361914; doi:10.1017/S0007114524000722)
Supplement: Luise et al. supplementary material [file S0007114524000722sup001.docx]

Manuscript title: Effect of different doses of camelina cake inclusion as a substitute of dietary soybean meal on growth performance and gut health of weaned pigs.

Diana Luise ^a^, Federico Correa ^a^, Giulia Cestonaro ^b^, Eleonora Sattin ^c^, Marcello Mele ^d^, Giuseppe Conte ^d^, Ivonne Archetti ^e^, Sara Virdis ^a^, Clara Negrini ^a^, Incoronata Galasso ^f^, Claudio Stefanelli ^g^, Maurizio Mazzoni ^h^, Luigi Nataloni ^b^, Paolo Trevisi ^a*^ & Enrico Costanzo ^b^

a *Department of Agricultural and Food Sciences (DISTAL)*, University of Bologna, Viale G Fanin, Bologna, 4127, Italy,

^b^ Cereal Docks S.p.A - Dipartimento Ricerca & Innovazione (E. Costanzo, G. Cestonaro), Cereal Docks S.p.A (L. Nataloni) via Innovazione 1, Camisano Vicentino, 36043, Italy

^c^ BMR genomics, Via della repubblica, Padova, 35131, Italy;

*^d^ Dipartimento di Scienze Agrarie, Alimentari, Agro-ambientali, Università di Pisa, Via del Borghetto, 80, 56124 Pisa, Italy*

*^e^ Istituto Zooprofilattico Sperimentale della Lombardia e dell’Emilia Romagna Bruno Ubertini, V. Bianchi 9, 25124Brescia, Italy;*

*^f^ CNR Istituto di Biologia e Biotecnologia Agraria (IBBA)*

*^g^ Department for Life Quality Studies, Alma Mater Stu‑ diorum, University of Bologna, 47921 Rimini, Italy*

*^h^ Department of Veterinary Science, University of Bologna, 46, 40064, Ozzano dell'Emilia, Italy*

**Supplementary Table 1**. Fatty acid profile of camelina cake and of the diets.

| Fatty acid | Camelina cake | CO | C4 | C8 | C12 |
| --- | --- | --- | --- | --- | --- |
| C4:0 | <0.05 | <0.05 | <0.05 | <0.05 | <0.05 |
| C6:0 | <0.05 | <0.05 | <0.05 | <0.05 | <0.05 |
| C7:0 | <0.05 | <0.05 | <0.05 | <0.05 | <0.05 |
| C8:0 | <0.05 | <0.05 | <0.05 | <0.05 | <0.05 |
| C10:0 | <0.05 | <0.05 | <0.05 | <0.05 | <0.05 |
| C10:1 | <0.05 | <0.05 | <0.05 | <0.05 | <0.05 |
| C11:0 | <0.05 | <0.05 | <0.05 | <0.05 | <0.05 |
| C12:0 | <0.05 | 0.12 | 0.13 | 0.10 | 0.08 |
| C13:0 | <0.05 | <0.05 | <0.05 | <0.05 | <0.05 |
| C14:0 | 0.07 | 0.57 | 0.65 | 0.59 | 0.54 |
| C14:1 | <0.05 | <0.05 | <0.05 | <0.05 | <0.05 |
| C15:0 | <0.05 | 0.06 | 0.07 | 0.06 | 0.07 |
| C15:1 | <0.05 | <0.05 | <0.05 | <0.05 | <0.05 |
| C16:0 | 6.40 | 19.10 | 21.10 | 18.20 | 15.20 |
| C16:1 (9t) | <0.05 | <0.05 | <0.05 | <0.05 | <0.05 |
| C16:1 (9c) | 0.13 | 0.39 | 0.44 | 0.45 | 0.44 |
| C16:2 (9c,12c) | <0.05 | <0.05 | <0.05 | <0.05 | <0.05 |
| C16:2 (7c, 10c) / w6 | <0.05 | <0.05 | <0.05 | <0.05 | <0.05 |
| C17:0 | 0.05 | 0.10 | 0.10 | 0.10 | 0.08 |
| C17:1 | <0.05 | <0.05 | <0.05 | <0.05 | <0.05 |
| C17:1 (9c) | <0.05 | 0.09 | <0.05 | <0.05 | <0.05 |
| C17:1 (10c) | <0.05 | <0.05 | <0.05 | <0.05 | <0.05 |
| C18:0 | 2.39 | 3.30 | 3.20 | 2.90 | 2.70 |
| C18:1 (t) | <0.05 | <0.05 | <0.05 | <0.05 | <0.05 |
| C18:1 (6c) | <0.05 | <0.05 | <0.05 | <0.05 | <0.05 |
| C18:1 (9c)/w9 | 15.40 | 25.90 | 25.60 | 23.20 | 21.10 |
| C18:1 (11c) | 1.19 | 1.07 | 0.97 | 1.00 | 1.02 |
| C18:2 (t) | 0.07 | 0.06 | 0.07 | 0.06 | 0.06 |
| C18:2 (9c, 12c) w6 | 21.10 | 42.30 | 35.50 | 34.70 | 35.00 |
| C18:3 (6c, 9c, 12c), w6 | <0.05 | <0.05 | <0.05 | <0.05 | <0.05 |
| C18:3 (9c, 12c, 15c), w3 | 30.60 | 3.70 | 6.20 | 9.80 | 12.70 |
| C18:3 (t) | 0.22 | <0.05 | <0.05 | 0.08 | 0.10 |
| C18:4 (6c, 9c, 12c, 15c) w3 | <0.05 | 0.18 | 0.20 | 0.21 | 0.21 |
| C20:0 | 1.46 | 0.37 | 0.50 | 0.64 | 0.75 |
| C20:1 (9c) | <0.05 | 0.05 | <0.05 | <0.05 | <0.05 |
| C20:1 (11c) | 13.10 | 0.72 | 2.22 | 3.90 | 5.20 |
| C20:3 (11c, 14cM 17c), w6 | 1.91 | <0.05 | 0.26 | 0.51 | 0.70 |
| C20:3 (11c, 14c, 17c) w3 | 1.19 | <0.05 | 0.16 | 0.31 | 0.43 |
| C20:3 (8c, 11c, 14c) w6 | <0.05 | 0.05 | <0.05 | <0.05 | <0.05 |
| C20:4 (5c, 8c, 11c, 14c), w6 | <0.05 | <0.05 | <0.05 | <0.05 | <0.05 |
| C20:4 (8c, 11c, 14c, 17c), w3 | <0.05 | <0.05 | <0.05 | <0.05 | <0.05 |
| C20:5 (5c, 8c, 11c, 14c, 17c) / w3 | <0.05 | 0.39 | 0.43 | 0.45 | 0.45 |
| C21:0 | <0.05 | <0.05 | <0.05 | <0.05 | <0.05 |
| C22:0 | 0.34 | 0.31 | 0.24 | 0.25 | 0.27 |
| C22:1 (11c) | <0.05 | 0.50 | 0.55 | 0.60 | 0.60 |
| C22:1 (13c) | 3.20 | <0.05 | 0.42 | 0.83 | 1.13 |
| C22:2 (13c, 16c) | 0.19 | <0.05 | <0.05 | <0.05 | <0.05 |
| C22:4 (7c, 10c, 13c, 16c) | <0.05 | <0.05 | <0.05 | <0.05 | <0.05 |
| C22:5 (4c,7c,10c,13c,16c) w6 | <0.05 | <0.05 | <0.05 | <0.05 | <0.05 |
| C22:5 (7c,10c,13c,16c,19c), w3 | <0.05 | <0.05 | <0.05 | <0.05 | <0.05 |
| C22:6(4c,7c,10c,13c,16c,19c), w3 | <0.05 | 0.56 | 0.65 | 0.63 | 0.68 |
| C23:0 | <0.05 | <0.05 | <0.05 | <0.05 | <0.05 |
| C24:0 | 0.20 | 0.19 | 0.17 | 0.19 | 0.20 |
| C24:1 (15c), w9 | 0.66 | <0.05 | 0.13 | 0.17 | 0.27 |
| Total saturated fatty acids | 10.90 | 24.10 | 26.20 | 23.00 | 19.90 |
| Total monounsaturated fatty acids | 33.70 | 28.70 | 30.30 | 30.10 | 29.80 |
| Total polyunsaturated fatty acids | 55.00 | 47.10 | 43.40 | 46.60 | 50.00 |
| Total trans fatty acids | 0.29 | 0.06 | 0.07 | 0.14 | 0.16 |
| Total Omega 3 fatty acids | 31.80 | 4.83 | 7.60 | 11.40 | 14.50 |
| Total Omega 6 fatty acids | 23.20 | 42.30 | 35.80 | 35.20 | 35.70 |
| Total Omega 9 fatty acids | 32.40 | 26.60 | 28.40 | 28.10 | 27.70 |

**Suppelementary Table 2.** Analysed chemical composition of the camelina cake and of the diets before the amino acid correction.

| Items | Camelina cake | CO | C4 | C8 | C12 |
| --- | --- | --- | --- | --- | --- |
| Crude protein | 28.40 | 17.48 | 16.74 | 17.06 | 16.89 |
| Fat | 4.64 | 5.71 | 5.78 | 5.49 | 5.53 |
| Cellulose |  | 4.21 | 4.78 | 4.31 | 5.02 |
| ADF | 15.00 | 4.00 | 5.50 | 5.40 | 6.10 |
| NDF | 21.30 | 16.80 | 18.70 | 17.60 | 17.90 |
| ADL | 3.50 | 2.10 | 0.90 | 1.40 | 1.30 |
| Ashes | 9.45 | 5.03 | 5.16 | 5.15 | 5.47 |
| Amino acids | | | | | |
| Lysine | 1.31 | 1.47 | 1.45 | 1.36 | 1.54 |
| Methionine | 0.48 | 0.42 | 0.42 | 0.39 | 0.46 |
| Cysteine (determined as cysteic acid) | 0.56 | 0.29 | 0.27 | 0.28 | 0.28 |
| Aspartic acid | 2.30 | 1.39 | 1.28 | 1.25 | 1.24 |
| Threonine | 1.14 | 0.81 | 0.82 | 0.74 | 0.86 |
| Serine | 1.23 | 0.73 | 0.69 | 0.69 | 0.69 |
| Glutamic acid | 4.74 | 3.27 | 3.14 | 3.13 | 3.13 |
| Proline | 1.48 | 1.10 | 1.09 | 1.08 | 1.08 |
| Glycine | 1.52 | 0.71 | 0.69 | 0.70 | 0.72 |
| Alanine | 1.29 | 0.76 | 0.73 | 0.73 | 0.73 |
| Valine | 1.45 | 0.89 | 0.90 | 0.97 | 1.07 |
| Isoleucine | 1.08 | 0.70 | 0.68 | 0.69 | 0.72 |
| Leucine | 1.83 | 1.27 | 1.23 | 1.26 | 1.31 |
| Tyrosine | 0.72 | 0.49 | 0.45 | 0.49 | 0.47 |
| Phenylalanine | 1.17 | 0.88 | 0.76 | 0.79 | 0.81 |
| Histidine | 0.68 | 0.45 | 0.45 | 0.46 | 0.50 |
| Arginine | 2.24 | 0.95 | 0.91 | 0.91 | 0.93 |
| Tryptophan | 0.32 | 0.23 | 0.24 | 0.24 | 0.24 |
| Total amino acids | 25.54 | 16.81 | 16.20 | 16.16 | 16.78 |

**Supplementary Table 3**. Gens, ID and TaqMan Assay ID used for the gene expression analysis.

| **Gene** | **Gene name** | **Assey Id** | **N.** |
| --- | --- | --- | --- |
|  |  |  | **assay TaqMan®** |
|  |  |  |  |
| *ZO-1* | Zonulin | Ss03373514_m1 | 4448892 |
| *OCLN* | Occludin | Ss03377507_u1 | 4331182 |
| *GPX2* | Glutathione Peroxidase | Ss03387478_u1 | 4448892 |
| *MyD88* | Innate Immune Signal Transduction Adaptor | Ss03389125_m1 | 4331182 |
| *TNF* | Tumor Necrosis Factor | Ss03391317_g1 | 4448892 |
| *HMBS* | Hydroxymethylbilane Synthase | Ss03388782_g1 | 4448491 |


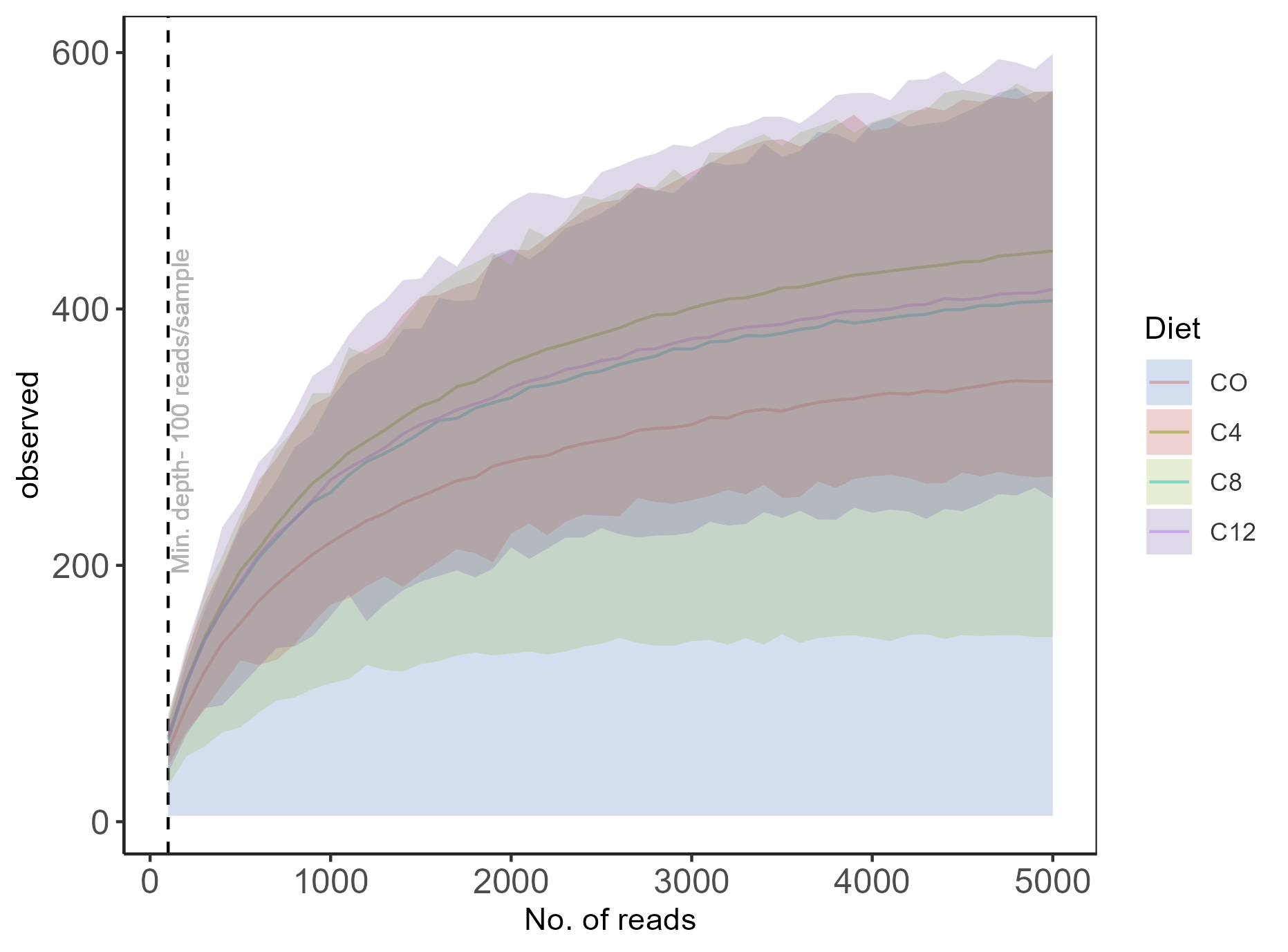


**Supplementary Figure 1**. Rarefaction curves of V3-V4 region of the 16s rRNA gene in faecal samples collected at d7 and d28 in piglets fed different doses of camelina cake. Diet*: CO = control diet; C4 = diet with the inclusion of 4% of CAM; C8 = diet with the inclusion of 8% of CAM; C12 = diet with the inclusion of 12% of CAM.
